# The need to differentiate at re‐engagement: lessons from South Africa and Zimbabwe's re‐engagement algorithms

**DOI:** 10.1002/jia2.26466

**Published:** 2025-07-07

**Authors:** Lynne S. Wilkinson, Helen Bygrave, Musa Manganye, Chiedza Mupanguri, Anna Grimsrud

**Affiliations:** ^1^ International AIDS Society Johannesburg South Africa; ^2^ Centre for Infectious Epidemiology and Research, Faculty of Health Sciences University of Cape Town Observatory South Africa; ^3^ International AIDS Society London UK; ^4^ National Department of Health ‐ South Africa HIV/AIDS and STI Pretoria South Africa; ^5^ Ministry of Health and Child Care Harare Zimbabwe; ^6^ International AIDS Society Cape Town South Africa

1

As HIV epidemics mature, effectively addressing interruptions in antiretroviral therapy (ART) becomes increasingly critical to reducing morbidity, mortality and transmission [1, 2, 3]. Prolonged disengagement from ART places significant demands on health systems, including the need to manage advanced HIV disease (AHD), higher rates of hospitalisation and preventable new HIV acquisitions.

Disengagement from HIV care is the result of individual, interpersonal and/or structural vulnerabilities combined with life disruptions, such as unexpected travel, that impact a person's ability to remain in care [4, 5]. Fortunately, many individuals are self‐motivated to return to care. However, their timely re‐engagement often depends on removing barriers and introducing valued facilitators [6, 7]. Data from Malawi and South Africa show that the majority of people attempt return within 3 months of missing a scheduled appointment, but more country‐specific time‐to‐return data is needed [8, 9].

Disengagement occurs across the HIV care cascade, with proportionally more people disengaging during early ART but greater numbers disengaging thereafter. In mature, generalised HIV epidemics, disengagement is common among all population groups, reinforcing the need for broad, scalable approaches that improve re‐engagement outcomes [3].

Re‐engagement involves two main intervention categories: tracing to encourage return, and enhancing the return experience to reduce interruption length and repeat disengagement [5]. This viewpoint focuses on the latter by removing barriers and adapting service delivery to support re‐engagement.

HIV programmes must first recognise that ART interruptions are common and prioritise facilitating easy, quick and sustained re‐engagement [3]. Some individuals fear returning due to concerns about disappointing healthcare workers and experiencing punitive actions [6, 10, 11]. Respectful care for returning clients can reduce fear and promote timely return. Re‐engagement guidance should emphasise same‐day ART provision, avoiding multiple visits [7] or transfer documentation collection [11]. Long waiting times and penalisation for missed appointments should be monitored and penalisation [6, 7]. People re‐engaging in care commonly previously struggled with frequent appointments, inconvenient locations and long wait times. Accelerating access to less‐intensive differentiated service delivery (DSD) can reduce client burden and help prevent future interruptions [6, 7]. Frequent clinical visits should be reserved for when clinically necessary.

Ministries of health are starting to implement guidance on managing people returning to care, focusing on respectful care and a shift away from one‐size‐fits‐all intensified clinical management, with its monthly appointments and multiple adherence counselling sessions. Differentiating care pathways identify individuals who are simply “late” for their scheduled visit, with no or only a brief treatment interruption, and who can continue routine care, including in DSD models. They also identify those who require further assessment. Two key assessments at return guide further differentiation. First, clinical stability, assessed through signs of opportunistic infections, mental health concerns, AHD or an elevated viral load prior to disengagement. Second, time since the missed appointment which indicates potential interruption duration and AHD risk.

To illustrate how these programmatic considerations have been applied, we highlight the national re‐engagement algorithms of South Africa and Zimbabwe—the first two countries to formalise differentiated re‐engagement pathways in national guidelines – offering valuable lessons for other settings. These are shown in Figure 1.

**Figure 1:**
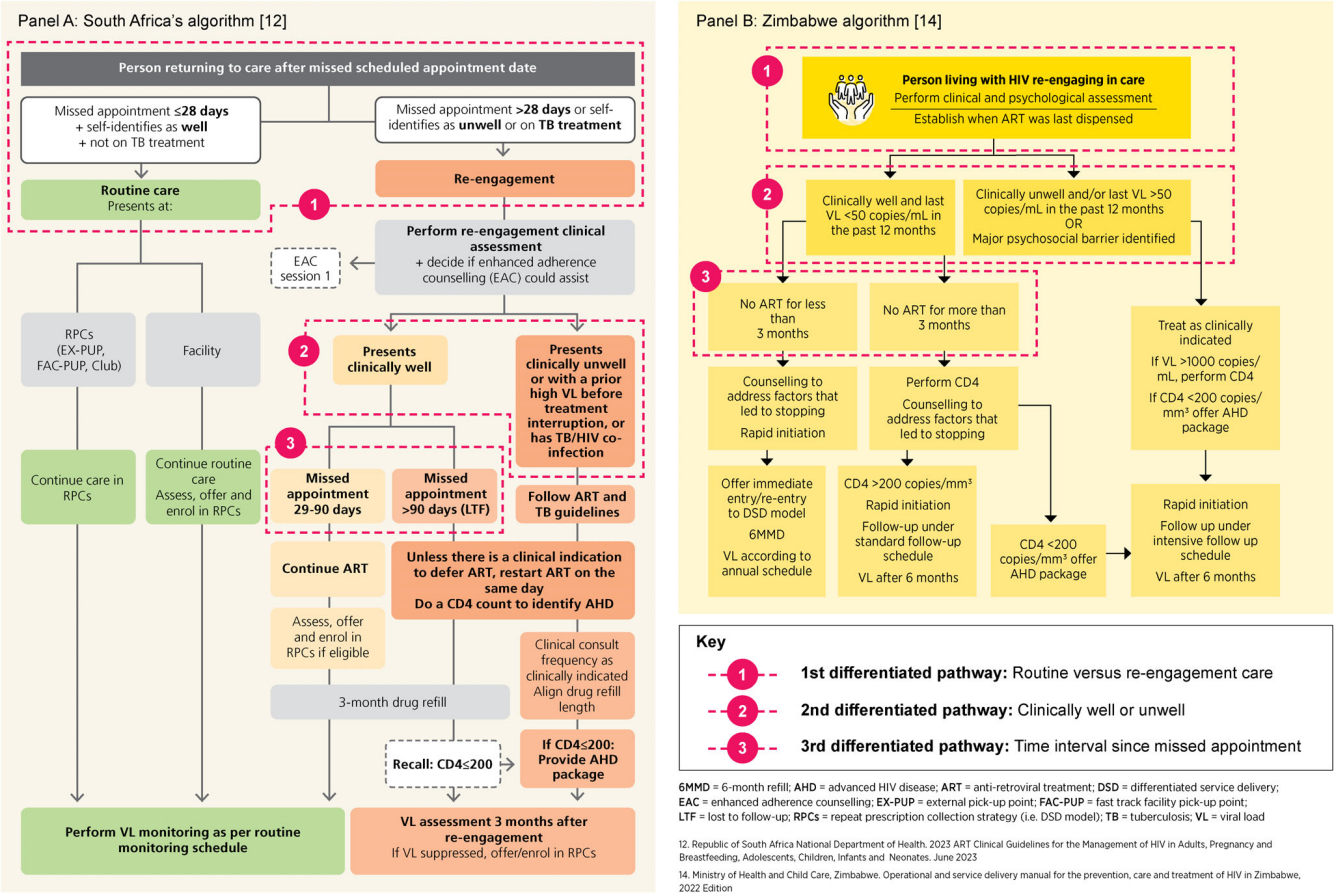
Algorithm‐based differentiated clinical management and service delivery pathways at re‐engagement.

South Africa defines re‐engagement as missing an appointment and returning to care unwell or by more than 28 days [12]. Their algorithm (Panel A) distinguishes routine from re‐engagement care [12]. People late by 28 days or less continue or enrol in DSD models. The viral load testing schedule remains unchanged. Those missing appointments for more than 28 days or self‐identifying as unwell undergo a clinical assessment and, unless clinically indicated, continue or restart ART on the same day. Clinically unstable individuals, regardless of interruption duration, require a repeat CD4 test to identify AHD and their follow‐up schedule is determined by need. Clinically stable individuals are assessed for time since the missed appointment, with CD4 testing required for those missing for more than 90 days. Those missing appointments by 29–90 days are managed similarly to individuals 28 days or less late. Both clinically unstable and “more than 90 days late” groups require a follow‐up viral load test after 3 months and a 3‐month ART refill unless earlier clinical care is needed. A month later, if suppressed, they are offered DSD enrolment.

Piloting an earlier version in nine health facilities revealed that engaged leadership, provider empathy and alignment with existing workflows were critical for successful adoption [13].

Zimbabwe defines re‐engagement as stopping ART after a missed visit. A clinical assessment is required for everyone re‐engaging to differentiate between those who are clinically stable and unstable (Panel B) [14]. For clinically stable individuals, those who are less than 3 months late are re‐initiated within 7 days and (re)enrolled in DSD models after receiving adherence counselling sessions. The viral load testing schedules remain unchanged. Clinically unstable individuals include those who are unwell, have an elevated viral load within the past 12 months or have significant psychosocial challenges. They require a follow‐up CD4 if the last viral load exceeded 1000 copies/ml, with their appointment schedule tailored to clinical needs. For all individuals more than 3 months late, CD4 counts are repeated to assess for AHD. Those with a CD4 count above 200 cells/mm^3^ follow the standard ART initiation schedule, with follow‐up clinical reviews after 1, 3 and 6 months—after which a viral load test is done. At the subsequent visit, suppressed individuals are offered DSD models, including 6‐month ART refills.

In an evaluation of the AHD screening component across 70 facilities, 23% of re‐engaging clients received CD4 testing, with 41% of those tested having CD4 < 200 cells/mm^3^. Staff shortages and commodity constraints posed challenges, particularly for point‐of‐care CD4 testing, while facilities trained on the use of the algorithm, conducted screening more confidently [15].

The South African and Zimbabwean algorithm‐based differentiated pathways for re‐engagement offer scalable approaches to facilitating efficient and durable return to care. By ensuring minimal disruption to individuals’ lives through accelerated access to extended ART refills and less‐intensive DSD, these approaches reduce the burden for clinically stable clients while ensuring necessary oversight for those with increased clinical needs, including AHD. Importantly, they ensure the re‐engagement process is person‐centred, with a focus on enhancing the return experience and mitigating barriers that may lead to prolonged or future interruptions. These adaptable approaches allow healthcare systems to address individual needs while optimising resources for broader population coverage.

## COMPETING INTERESTS

The authors declare that they have no conflict of interest.

## AUTHORS’ CONTRIBUTIONS

The concept for this commentary was developed by LSW, HB and AG. LSW wrote the first draft. All authors contributed and approved the final version.

## Data Availability

Data sharing is not applicable to this article as no datasets were generated or analysed during the current study.
